# Primary pulmonary histiocytic sarcoma with high PD‐L1 expression benefited from immunotherapy: A case report and bioinformatic analysis

**DOI:** 10.1111/crj.13741

**Published:** 2024-03-07

**Authors:** Yuanjie Lin, Qian Cao, Aonan Hong, Xiao Liang

**Affiliations:** ^1^ Department of Respiratory Medicine Jiangnan University Medical Center Wuxi China; ^2^ Department of Anesthesiology Jiangnan University Medical Center Wuxi China; ^3^ Affiliated Hospital of Nanjing University of Chinese Medicine Jiangsu Province Hospital of Chinese Medicine Nanjing China

**Keywords:** immunotherapy, nivolumab, PD‐L1, pembrolizumab, primary pulmonary histiocytic sarcoma

## Abstract

Histiocytic sarcoma is an aggressive haematopoietic malignancy accounting for less than 1% of haematolymphoid neoplasms with a diagnosis based on morphology and immunophenotype of tissue biopsies with a very poor prognosis. Here, we report a 45‐year‐old man who was diagnosed with primary pulmonary histiocytic sarcoma with systemic metastases, with partial remission (PR) treated with cyclophosphamide, doxorubicin, vincristine and prednisone (CHOP) chemotherapy, but it relapsed soon after therapy above. Tests demonstrated that TMB was 21 Muts/Mb PD‐L1 expression was 90% positive, and the disease has been well‐controlled over 3 years using immune checkpoint inhibitors (nivolumab and pembrolizumab). Bioinformatic pan‐cancer analysis verified that there was the highest genetic alteration frequency of PD‐L1 in which amplification accounted for the majority of sarcoma tumour samples. Following that, we found that the genetic alteration of PD‐L1 was associated with poor prognosis in sarcoma patients in terms of overall survival (OS) (*p* = 1.51 × 10^−4^), progress‐free survival (PFS) (*p* = 4.90 × 10^−2^) and disease‐specific survival (DSS) (*p* = 4.90 × 10^−2^). To our knowledge, this may be the first reported case with high PD‐L1 expression in primary pulmonary histiocytic sarcoma who may benefit from immunotherapy such as nivolumab and pembrolizumab significantly and safely.

## Graphical abstract

This may be the first reported case with high PD‐L1 expression in primary pulmonary histiocytic sarcoma who may benefit from immunotherapy. Routine PD‐L1 expression detection is recommended for histiocytic sarcoma patients especially those who got advanced cancer. Patients with high PD‐L1 expression in primary pulmonary histiocytic sarcoma may benefit from immunotherapy such as nivolumab and pembrolizumab significantly and safely.

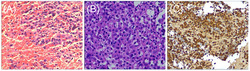

## 1 INTRODUCTION

Histiocytic sarcoma is an extremely rare but highly aggressive haematopoietic malignancy in which tumour cells exhibit morphological and immunophenotypic features of histiocytic differentiation.^1^ The overall incidence of histiocytic sarcoma was only 0.17/1 000 000, but patients with histiocytic sarcoma have poor median overall survival (OS) of only 6 months.^2^ Although the aetiology is unknown, histiocytic sarcoma may result from prior low‐grade B‐cell lymphomas by transdifferentiation, such as small lymphocytic lymphoma, marginal zone lymphoma and follicular lymphoma.^3^, ^4^, ^5^ Histologically, this tumour generally shows large, round or ovoid pleomorphic cells with abundant eosinophilic cytoplasm, oval or irregular nuclei, vesicular chromatin and large nucleoli,^6^ while some are dominated by spindle cells.^7^, ^8^, ^9^, ^10^ Still, immunohistochemistry of histiocytic sarcoma shows the expressions of histiocytic markers, including CD68, CD163 and lysozyme, with the absence of B‐cell, T‐cell, dendritic cell, epithelioid cell, myeloid cell markers.

The most common predilection sites are skin and connective tissue, followed by lymph nodes, respiratory system, nervous system, gastrointestinal tract, bone marrow and haematopoietic system, spleen and reticuloendothelial system^2^; however, pulmonary presentation is rare.^6^, ^11^ Patients with early and localized disease usually undergo surgical resection or radical radiation therapy, whereas patients with advanced disease typically receive multi‐drug chemotherapy.^6^, ^8^ For advanced histiocytic sarcoma, the most commonly used chemotherapy regimen is clophosphamide, doxorubicin, vincristine and prednisone (CHOP); other choices mainly include ifosfamide, carboplatin and etoposide (ICE); prednisone, methotrexate, doxorubicin, cyclophosphamide, etoposide, mechlorethamine, vincristine and procarbazine (ProMACE‐MOPP); and cladribine, cytarabine, G‐CSF and mitoxantrone (CLAG‐M)^6^, ^12^; however, most patients with histiocytic sarcoma treated with chemotherapy had a short OS. Targeted therapy for histiocytic sarcoma may be a good choice, but the efficacy of the targeted drugs needs clinical trials to be confirmed, and better treatment options are still being explored.

With the development of immunotherapy, PD‐L1 expression has been extensively detected in histiocytic sarcoma patients with a significant proportion having different degrees of PD‐L1 expression.^13^, ^14^, ^15^ As far as we know, only three patients with high PD‐L1 expression in histiocytic sarcoma, but not primary pulmonary, have received immunotherapy with nivolumab, but the efficacies are different. So, our report may be the first reported case of primary pulmonary histiocytic sarcoma with high PD‐L1 expression which has been significantly relieved after immunotherapy.

## 2 CASE PRESENTATION

A 45‐year‐old male was found to right lung lesions with multiple lymph nodes in mediastinum, right supraclavicular region and both sides of iliac wings lesions in December 2017 when he presented with a cough. The patient had a history of hypertension and type 2 diabetes mellitus, a history of penicillin allergy, no smoking or alcohol habits and no family history. Biopsies performed by fine needle aspiration (FNA) of lymph nodes in December 2017, May 2018 and June 2018, respectively, were non‐diagnostic. On 26 December 2018, surgical biopsies of both alliums and cervical lymph nodes confirmed histiocytic sarcoma after a pathology consultation with Zhongshan Hospital Affiliated with Fudan University. Pathology showed a malignant tumour with necrosis, pleomorphic cells, abundant cytoplasm and easily visible mitoses. Immunohistiochemistry showed CD63+, CD163+, langerin–, CD1a–, CD3–, CD20–, CD21–, CD23–, CD34–, S‐100–, AE1/AE3– and Ki‐67+ (50%) (Table 1). Bone marrow aspiration showed no abnormalities in bone marrow smear, lymphoma immunophenotyping or fusion genes.

**TABLE 1:** Immunohistochemistry of the HS patient by surgical biopsy specimens.

Antibodies	Results
CD63	+
CD163	+
Langerin	−
CD1a	−
CD3	−
CD20	−
CD21	−
CD23	−
S‐100	−
CD34	−
AE1/AE3	−
Ki‐67	+(50%)

**FIGURE 1:**
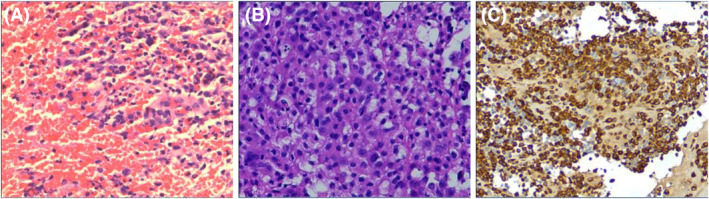
Pathological features of histiocytic sarcoma. Stained with haematoxylin and eosin: (A) pathology of right upper lobe biopsy specimen, showing necrosis infiltrated with abundant degenerative eosinophil‐like cells and histiocyte‐like cells; (B) paraffin block pathology of pleural fluid cells, showing tumour cells. (C) Immunostaining was focally positive for CD68. Magnification, 400×.

The patient received six cycles of CHOP chemotherapy from 23 January 2019 and achieved PR initially. The disease recurred leading to his admission to hospital with severe symptoms including shortness of breath, palpitations and night sweats on 13 September 2019. Bronchoscopic intervention and drainage of pleural effusion were undergone to relieve the symptoms. Pathology of the right upper lobe and pleural fluid were consistent with histiocytic sarcoma (Figure 1). Meanwhile, the surgical biopsy specimens were sent to UT MD Anderson Cancer Center for consultation. The tests demonstrated that PD‐L1 expression was 90% positive, tumour mutational burden (TMB) was 11.6 Muts/Mb and genes detected were negative. Based on the high expression of PD‐L1, this patient received nivolumab 240 mg (3 mg/kg) every 2 weeks from 23 October 2019.

**FIGURE 2:**
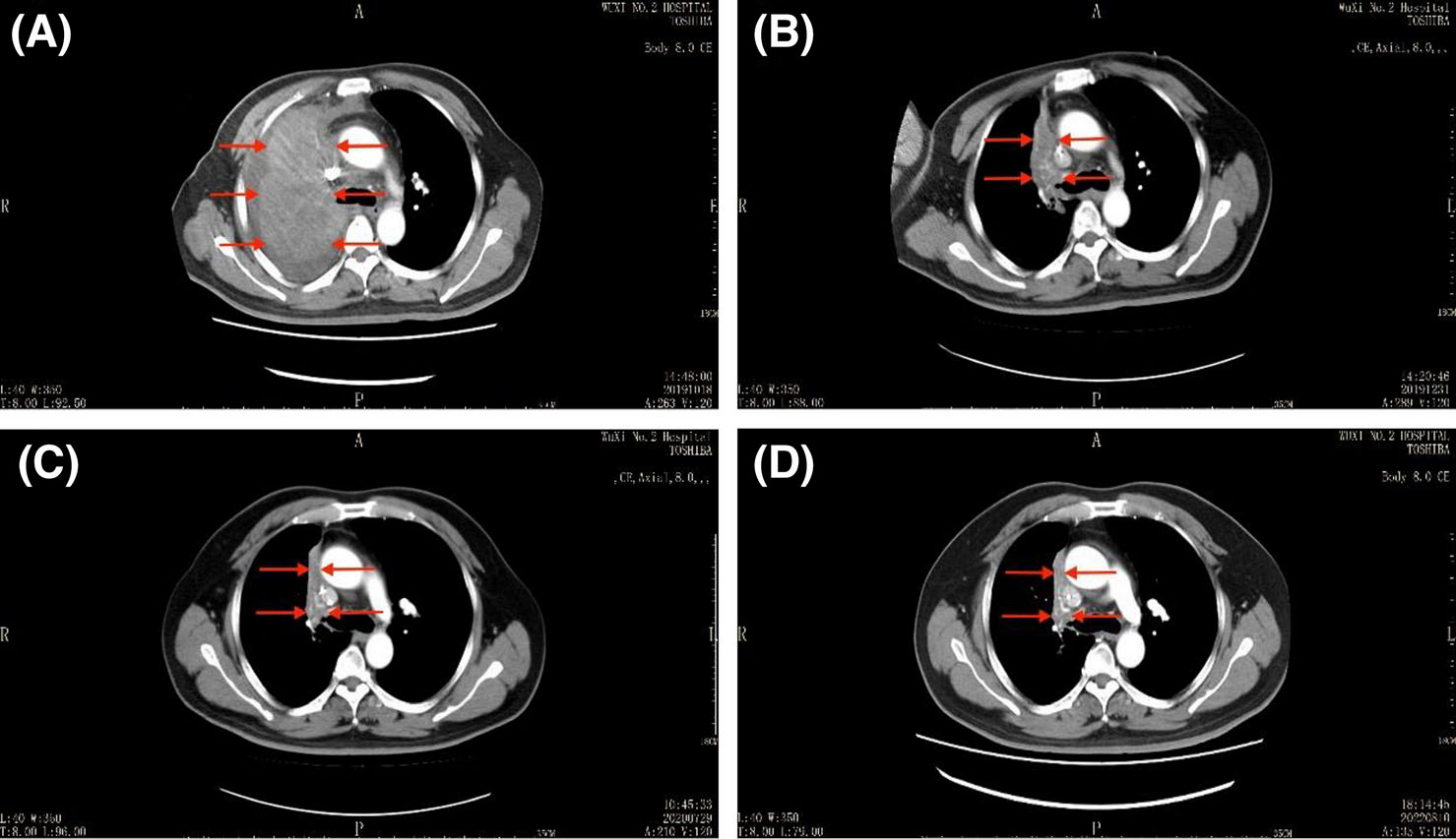
Contrast‐enhanced computed tomography (CT) scans of the histiocytic sarcoma patient before and after immunotherapy, showing the lesions were significantly reduced and remained stable for nearly 3 years. (A) 18 October 2019, 141 × 77 mm; (B) 31 December 2019, 90 × 24 mm; (C) 29 July 2020, 72 × 12 mm; (D) 10 August 2022, 70 × 8 mm.

**FIGURE 3:**
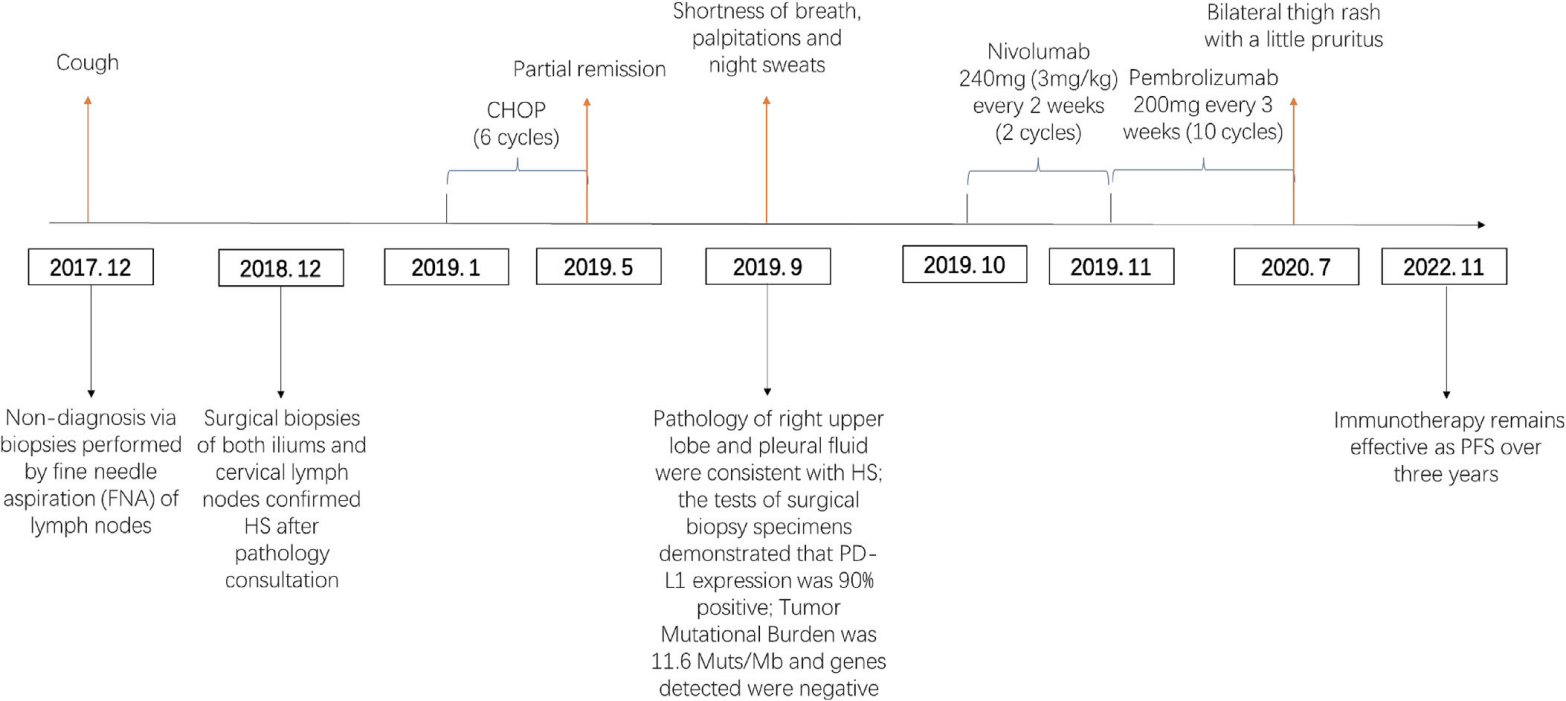
With only cough symptoms, the patient was not diagnosed definitively in December 2017, surgical biopsies of both iliums and cervical lymph nodes confirmed histiocytic sarcoma after pathology consultation in December 2018, then the patient underwent CHOP (six cycles) in January 2018; the patient received nivolumab 240 mg (3 mg/kg) every 2 weeks (two cycles) from October 2019, then with pembrolizumab 200 mg every 3 weeks (10 cycles) to July 2020, immunotherapy remains effective as PFS over 3 years.

The lesions significantly reduced, and symptoms gradually disappeared after immunotherapy (Figure 2). On 26 November 2019, the tests of these specimens sent to another laboratory showed that TMB was 21 (>20) Muts/Mb, microsatellite status (MS) was stable and genomic findings revealed CD274 (PD‐L1) (amplification), JAK1 (Y652H), PDCD1LG2 (PD‐L2) (amplification), TET2 (R1465), BTG1 (Q36H), MSH3 (splice site 1453 + 2 T > A), PIM1 (K24N, amplification) and SOCS1 (G122fs*125, rearrangement exon 2) (Table 2). Genomic results did not lead to the implementation of other treatments due to the significant efficacy of immunotherapy. After comparing the prices and convenience, nivolumab was changed to pembrolizumab 200 mg every 3 weeks after 10 cycles. Adverse events included bilateral thigh rash with a little pruritus which resolved spontaneously. So far, the patient underwent 12 times of immunotherapy which remains effective with a follow‐up of over 3 years (Figure 3).

**TABLE 2:** Genomic findings of the HS patient by surgical biopsy specimens on 26 November 2019.

Items	Results
TMB	21 Muts/Mb
MS	Stable
CD274 (PD‐L1)	Amplification
JAK1	Y652H
PDCD1LG2 (PD‐L2)	Amplification
TET2	R1465
BTG1	Q36H
MSH3	Splice site 1453 + 2 T > A
PIM1	K24N, amplification
SOCS1	G122fs*125, rearrangement exon 2

**FIGURE 4:**
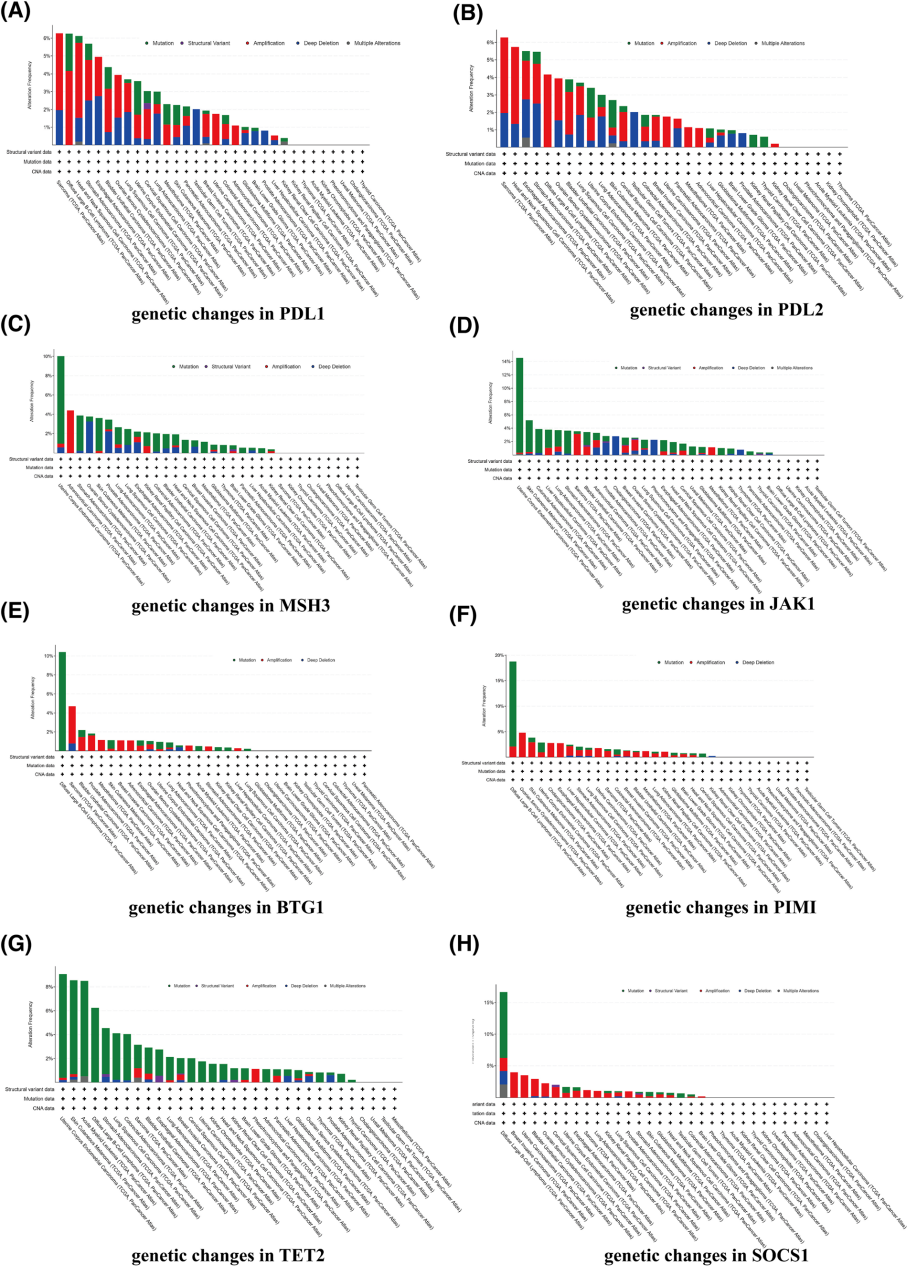
Genetic alteration in various tumour types of TCGA analysed by cBioPortal. Genetic alterations of (A) PD‐L1, (B) PD‐L2, (C) MSH3, (D) JAK1, (E) BTG1, (F) PIM1, (G) TET2 and (H) SOCS1 in pan‐cancer.

**FIGURE 5:**
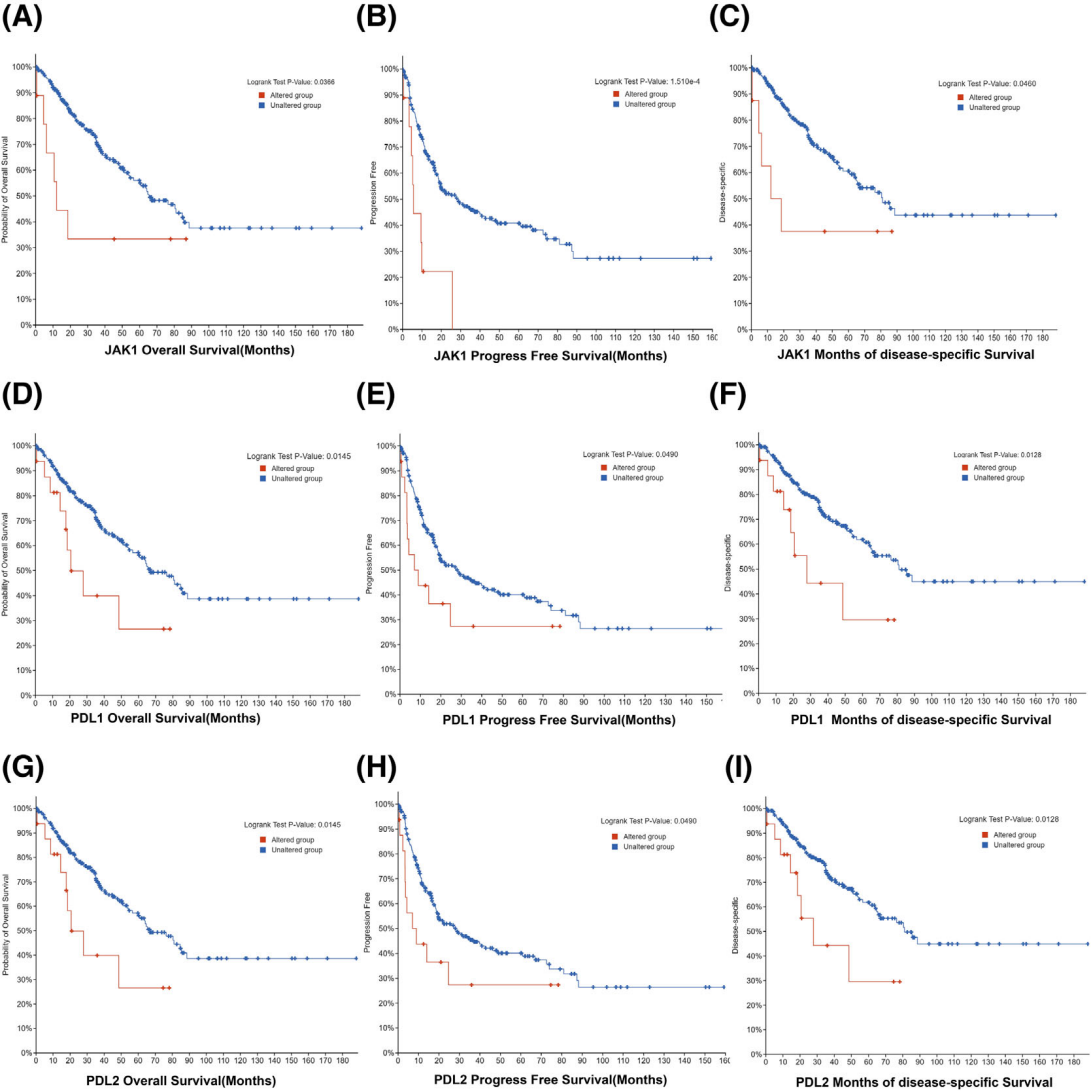
The correlations between genes alterative status and OS, PFS and DSS of patients with sarcoma analysed by cBioPortal. The correlations between (A) OS, (B) PFS, (C) DSS and JAK1 alterative status; between (D) OS, (E) PFS, (F) DSS and PD‐L1 alterative status; between (G) OS, (H) PFS, (I) DSS and PD‐L2 alterative status.

The cBio Cancer Genomics Portal (CBioPortal, https://www.cbioportal.org/) was utilized to analyse PD‐L1, PD‐L2, MSH3, JAK1, BTG1, PIM1, TET2 and SOCS1 genetic alterations in pan‐cancer. The tool provided information on the frequency of gene mutations and copy number changes in various cancer types which were calculated by ‘Cancer Types Summary’ module base on TCGA pan‐cancer datasets. There were the highest PD‐L1, PD‐L2 genetic alteration frequency in which amplification accounted for the majority in sarcoma tumour samples with the highest genetic amplification frequencies of JAK1 and BTG1 (Figure 4). The relationship between gene alteration status and prognosis in sarcoma was examined by dividing cases into categories based on molecular profiles and generating a survival plot based on the presence of copy number alterations (altered and unaltered groups). Following that, we found that the genetic alterations of JAK1, PD‐L1 and PD‐L2 in those eight genes mentioned above were associated with poor prognosis in sarcoma patients in terms of OS (*p* = 3.66 × 10^−2^; *p* = 1.51 × 10^−4^; *p* = 4.60 × 10^−2^), progress‐free survival (PFS) (*p* = 1.45 × 10^−2^; *p* = 4.90 × 10^−2^; *p* = 1.28 × 10^−2^) and disease‐specific survival (DSS) (*p* = 1.45 × 10^−2^; *p* = 4.90 × 10^−2^; *p* = 1.28 × 10^−2^) (Figure 5).

## 3 DISCUSSION

Histiocytic sarcoma is an exceedingly rare haematopoietic malignancy which might be difficult to diagnose. The diagnostic process in this patient was very hard. Multiple fine‐needle aspiration biopsies were all negative for pathology. The diagnosis took 1 year and was finally confirmed by surgical biopsy specimens. The demonstration of histiocytic markers (CD68 and CD163) and the absence of Langerhans cell markers (langerin and CD1a), T‐cell marker (CD3), B‐cell markers (CD20), mature B‐cell and follicular dendritic cell marker (CD21), B‐cell chronic lymphocytic leukaemia and lymphoproliferative disease marker (CD23) supported the diagnosis of histiocytic sarcoma.

Although the clinical presentation and sites of involvement are different, the prognosis is often poor and OS remains short due to high malignancy of the disease. For metastatic histiocytic sarcoma cases, chemotherapy is the most commonly used treatment, especially the CHOP regimen. The patient was initially treated with a CHOP regimen and achieved PR, but the disease progressed about 7 months later. Genomic findings in a large cohort of histiocytic sarcoma using a targeted next‐generation sequencing (NGS) approach identified driver alterations that involved the RAS‐MAPK signalling pathway (MAP 2K1, KRAS, NRAS, BRAF, PTPN11, NF1 and CBL) (57%), the PI3K signalling pathway (PTEN, MTOR, PIK3R1 and PIK3CA) (21%) and the tumour‐suppressor gene CDKN2A (46%).^16^ With TMB > 20 Muts/M and a PD‐L1 gene amplification, there is some efficacy shown for targeted therapy,^17^, ^18^, ^19^ which is consistent with our results, this case with TMB 21 (>20) Muts/Mb and CD274 (PD‐L1) amplification benefited from immunotherapy.

In recent years, PD‐L1 expression has been frequently found in patients with sarcoma, which also was proved in our research, even with lower survival rates with higher genetic alterative frequency. Three cases of histiocytic sarcoma patients with PD‐L1 expression receiving nivolumab as treatment have been reported. The first case was a 66‐year‐old male with refractory histiocytic sarcoma who was treated with nivolumab after knowing PD‐L1 expression in 15% to 20% of the tumour. After three doses of nivolumab 3 mg/kg every other week, progressive disease was documented on positron emission tomography–computed tomography (PET‐CT).^13^ In another case with histiocytic sarcoma who was treated with nivolumab after noting PD‐L1 expression in 75% of the tumour got a pseudoprogression after using it for 2 months. She continued improvement by continuing to use it in the next 11 months with a PFS not reached.^14^ The third case was a 43‐year‐old female with refractory and relapsed histiocytic sarcoma whose PD‐L1 expression level of tumour cells was 75%. All metastatic lesions were in remission except for the primary site in the femoral head after administering nivolumab 200 mg biweekly for 12 cycles. She underwent primary site resection and left femoral head replacement surgery and received additional nivolumab treatment as consolidation therapy for 1 year.^15^ So, based on the latter two cases, we could find that there might be a certain effect of PD‐L1‐targeted therapy on histiocytic sarcoma with high PD‐L1 expression.

Our patient was critically ill due to disease recurrence after chemotherapy. With the help of bronchoscopic intervention and drainage of pleural effusion, the patient got the chance of further treatment as the consultation with the University of Texas MD Anderson Cancer Center took more than 1 month. PD‐L1 expression was detected as 90% positive, and immune checkpoint inhibitor nivolumab was applied after consultation; fortunately, the efficacy of immunotherapy in this patient was significant. These four histiocytic sarcoma patients who received immunotherapy had different results; nevertheless, none of them used the targeted therapy as first‐line therapy. It is speculated that the responses to immune checkpoint inhibitors may be related to the expression levels of PD‐L1, with higher expression leading to better results. More cases, as well as clinical studies, are needed to confirm whether immunotherapy can be used as first‐line therapy in histiocytic sarcoma patients and whether immunotherapy is superior to other treatments in primary pulmonary histiocytic sarcoma patients with high PD‐L1 expression.

From the results of bioinformatics, we found that there was the highest PD‐L1 genetic alteration frequency in which amplification accounted for the majority of sarcoma tumour samples, and we found that the genetic alterations of PD‐L1 were associated with poor prognosis in sarcoma patients in terms of OS, PFS and DSS, which showed the PD‐L1 gene was closely related to the occurrence and development of sarcoma. The above conclusions further confirmed that the administration of immunotherapy based on the PD‐L1 expression might be reasonable and effective for sarcoma patients.

In conclusion, routine PD‐L1 expression detection is recommended for histiocytic sarcoma patients especially those who have advanced cancer. Patients with high PD‐L1 expression in primary pulmonary histiocytic sarcoma may benefit from immunotherapy such as nivolumab and pembrolizumab significantly and safely. Then, more clinical studies are needed to identify the treatment efficacy of immunotherapy based on the expression of PD‐L1 on primary pulmonary histiocytic sarcoma.

## AUTHOR CONTRIBUTIONS

Xiao Liang designed the study. Yuanjie Lin collected the clinical data. Qian Cao participated in collecting the gene data. Aonan Hong and Yuanjie Lin analysed the data. Qian Cao reviewed and analysed data. Yuanjie Lin drafted the manuscript. Aonan Hong supervised the entire study. All authors contributed to the article and approved the submitted version.

## CONFLICT OF INTEREST STATEMENT

The authors declare that the research was conducted in the absence of any commercial or financial relationships that could be construed as a potential conflict of interest.

## ETHICS STATEMENT

The studies involving human participants were reviewed and approved by Nanjing University Medical Center. Written informed consent for participation was not required for this study under the national legislation and the institutional requirements. Written informed consent was obtained from the individual(s) for the publication of any potentially identifiable images or data included in this article.

## PUBLISHER'S NOTE

All claims expressed in this article are solely those of the authors and do not necessarily represent those of their affiliated organisations or those of the publisher, the editors and the reviewers. Any product that may be evaluated in this article, or claim that may be made by its manufacturer, is not guaranteed or endorsed by the publisher.

## Data Availability

The original contributions presented in the study are included in the article/Supporting Information. Further inquiries can be directed to the corresponding author.
